# Complete Genome Sequence of Mycoplasma gallisepticum Strain KUVMG001, an Isolate from South Korea

**DOI:** 10.1128/MRA.00331-21

**Published:** 2021-05-13

**Authors:** Yongjun Song, Taesoo Kim, Tae-Min La, Hong-Jae Lee, Yoonsuk Lee, Hyunjin Shin, Gyuhee Ahn, Won Hur, Seung-un Song, Joong-Bok Lee, Seung-Yong Park, In-Soo Choi, Sang-Won Lee

**Affiliations:** a College of Veterinary Medicine, Konkuk University, Seoul, Republic of Korea; University of Maryland School of Medicine

## Abstract

Mycoplasma gallisepticum causes chronic respiratory diseases in poultry and economic losses to the chicken and turkey industry. We report the complete genome sequence of the field isolate strain KUVMG001 of Mycoplasma gallisepticum from South Korea.

## ANNOUNCEMENT

Mycoplasma gallisepticum is an avian pathogen that causes chronic respiratory disease, decreased egg production, and decreased feed efficiency in poultry, which results in economic damage to the industry (1). Despite its importance, no complete genome sequence of Mycoplasma gallisepticum from South Korea has been announced.

The KUVMG001 strain of Mycoplasma gallisepticum is an isolate from a tracheal and cleft palate swab sample from a chicken with no clinical signs in South Korea. The sample was collected for a safety test of a Mycoplasma synoviae vaccine. The swab specimen was suspended in 5 ml of modified Frey’s broth (2). The broth suspension was filtered through a 0.45-μm syringe filter and then incubated at 37°C until the broth changed to an orange-yellow color. When the color change occurred, the broth was diluted by a 10-fold serial dilution method and then inoculated on modified Frey’s agar. To purify the isolate, three consecutive rounds of colony picking using Pasteur pipettes were performed.

A pure isolate of Mycoplasma gallisepticum was cultured in modified Frey’s broth at 37°C. After incubation, the broth culture was centrifuged at 17,500 × *g* for 10 min, and the pellet was resuspended in fresh modified Frey’s broth and cultured again to increase the number of *Mycoplasma* cells. Fifty milliliters of cultured broth was used for DNA preparation.

Illumina sequencing and Nanopore sequencing were used to obtain the complete genome (3). Genomic DNA for the Illumina sequencing was extracted using the QIAamp DNA minikit (Qiagen). The TruSeq Nano DNA kit (Illumina) was used for library preparation, according to the manufacturer’s instructions. The Illumina sequencing was performed using a HiSeq 4000 system, resulting in 101-bp, paired-end reads. FastQC software v0.11.7 was used for quality control. The total yield was 14,129,120 reads (proportion with a score of >Q30, 92.39%).

DNA for Nanopore sequencing was extracted using the MagAttract HMW DNA kit (Qiagen) according to the manufacturer’s instructions. No shearing or size selection of the DNA was performed. The Nanopore sequencing was performed following the native barcoding genomic DNA protocol (with EXP-NBD104, EXP-NBD114, and SQK-LSK109). The third barcode from the EXP-NBD104 kit and the SQK-LSK10 ligation kit were used. Sequencing was performed with a flow cell type R9.4.1 on the MinION Mk1B (Oxford Nanopore Technologies, Oxford, UK) for 48 h. Albacore v2.3.1 was used for base calling. Default parameters were used for all software unless otherwise specified. The output of Nanopore sequencing was 2.5 Gb (4 × 10^5^ reads; read *N*_50_, 12,694 bp; proportion with a score of >Q10, 77.9%).

Nanopore sequencing reads were subsampled with 100× coverage using Rasusa v0.3.0 (4) (read *N*_50_, 12,784 bp; reference genome size, 0.99 Mb). The subsample was assembled using Flye v2.7.1 (5). The assembled draft genome was polished with the Illumina sequencing reads using unicycler-polish, which is part of the Unicycler v.0.4.7 pipeline (6). Circularization of the assembled genome was double checked by visualization using Bandage v0.8.1 (7).

The complete genome sequence was annotated by the NCBI Prokaryotic Genome Annotation Pipeline (PGAP). The size of the genome was 1,009,936 bp with a GC content of 31.6%, with 760 coding sequences, 41 RNAs, and 60 pseudogenes.

### Data availability.

This whole-genome sequence with the annotation data is available in the NCBI database under the accession number CP070622, BioSample number SAMN16879834, and BioProject number PRJNA680431. SRR13148778 and SRR13148779 are the accession numbers for long-read and short-read data, respectively, in the NCBI SRA.
